# An unusual case of neurothekeoma of the arm in an adult

**DOI:** 10.1007/s10195-015-0386-3

**Published:** 2015-11-14

**Authors:** Federica Bergamin, Ezio Nicola Gangemi, Claudia Cerato, Alessandra Clemente, Marco Borsetti, Adolfo Suriani, Stefano Taraglio

**Affiliations:** 1 Department of Surgical Activities, Division of Plastic Surgery and Hand Surgery, Maria Vittoria Hospital, ASL TO2, Via Cibrario 72, 10144 Turin, Italy; 2 Department of Laboratory Diagnostics, Division of Pathology, Maria Vittoria Hospital, ASL TO2, Via Cibrario 72, 10144 Turin, Italy

**Keywords:** Soft tissue tumors, Myxoid tumors, Nerve sheath tumors, Hand surgery, Rare tumors

## Abstract

Neurothekeomas are uncommon benign neoplasms with a peripheral nerve sheath origin. This tumor usually involves dermis and is described as a small, solitary, slow growing and reddish to flesh-colored nodule or papule. Neurothekeoma preferentially affects the central aspect of the face, the arms or shoulders of women in the second and third decades of life. This is the first case report of neurothekeoma involving the wrist developing from synovial tissue and with uncertain clinical behavior in an adult female. The tumor was completely excised under brachial plexus block. Histopathologically, the examination of the microscopic slides revealed the presence of a 20-mm diameter, well-circumscribed and multilobulated tumor composed of abundant myxoid stroma with cellular elements; with immunohistochemistry there was positivity to vimentin but S100-protein, epithelial membrane antigen, cytokeratin AE1-3, CD99 and CD34 were all negative. This pattern suggested a myxoid tumor form of neurothekeoma, mixed subtype. The patient had an atypical local recurrence and was re-operated after 3 months. After 12 months there was no evidence of clinical recurrences confirmed by magnetic resonance evaluation. Basically, our case report adds an important element in the correct clinical management of neurotecheomas: faced with a histological diagnosis with an unusual localization and mixed or hypercellular type, clinicians must consider the possibility of an early local recurrence, suggesting a close clinical and radiological follow-up.

## Introduction

Neurothekeomas, or nerve sheath myxomas, are uncommon benign neoplasms with a peripheral nerve sheath origin [1, 2]. This tumor usually involves dermis or, less frequently, mucosal or submucosal tissue and, typically, is described as a small, solitary, slow growing and reddish to flesh-colored nodule or papule [3]. Neurothekeoma preferentially affects the central aspect of the face, the arms or shoulders of women in the second and third decades of life [4, 5]. To the best of our knowledge, this is the first case report of neurothekeoma involving the wrist developing from synovial tissue and with uncertain clinical behavior in an adult female.

## Case report

A 30-year-old woman presented with a large dorso-lateral synovial cyst of the right wrist previously ultrasonographically checked (2 months before); at the first examination the wrist was painful and swollen without a specific localization and the overlying skin appeared without local erythema. There was no reported fever, palpitations, irritability, dysphagia, dyspnea, weight loss, or other significant family medical history. The patient did not have a history of smoking but reported a history of recent trauma. The patient was treated with a functional bandage, rest and anti-inflammatory drugs for a week. At the next examination, there was an appreciable reduction of reactive synovitis of the extensor compartment, with evidence of the synovial cyst previously described. Therefore, an excision of the entire cyst was proposed.

Under brachial plexus block an S italic skin incision was performed on the dorso-ulnar surface of the wrist; the deep dissection showed a circumscribed and well-cleavable lesion like a tenosynovitis of the extensor carpi ulnaris (ECU) without a pedicle joint that was removed en bloc. Surprisingly, the pathologist found a 20-mm diameter, well-circumscribed and multilobulated tumor: each lobule was composed of abundant myxoid stroma with cellular elements in part fusal, in part epithelial and in part giant; all lobules showed a tendency to confluence but there was no cellular polymorphism or nuclear hyperchromia. Margins were tumor free. With immunohistochemistry there was positivity to vimentin but S100-protein, epithelial membrane antigen (EMA), cytokeratin AE1-3, CD99 and CD34 were all negative. There was a mitotic activity of 7 % (Ki67). This pattern suggested a myxoid tumor form of neurothekeoma, mixed subtype.

**Fig. 1:**
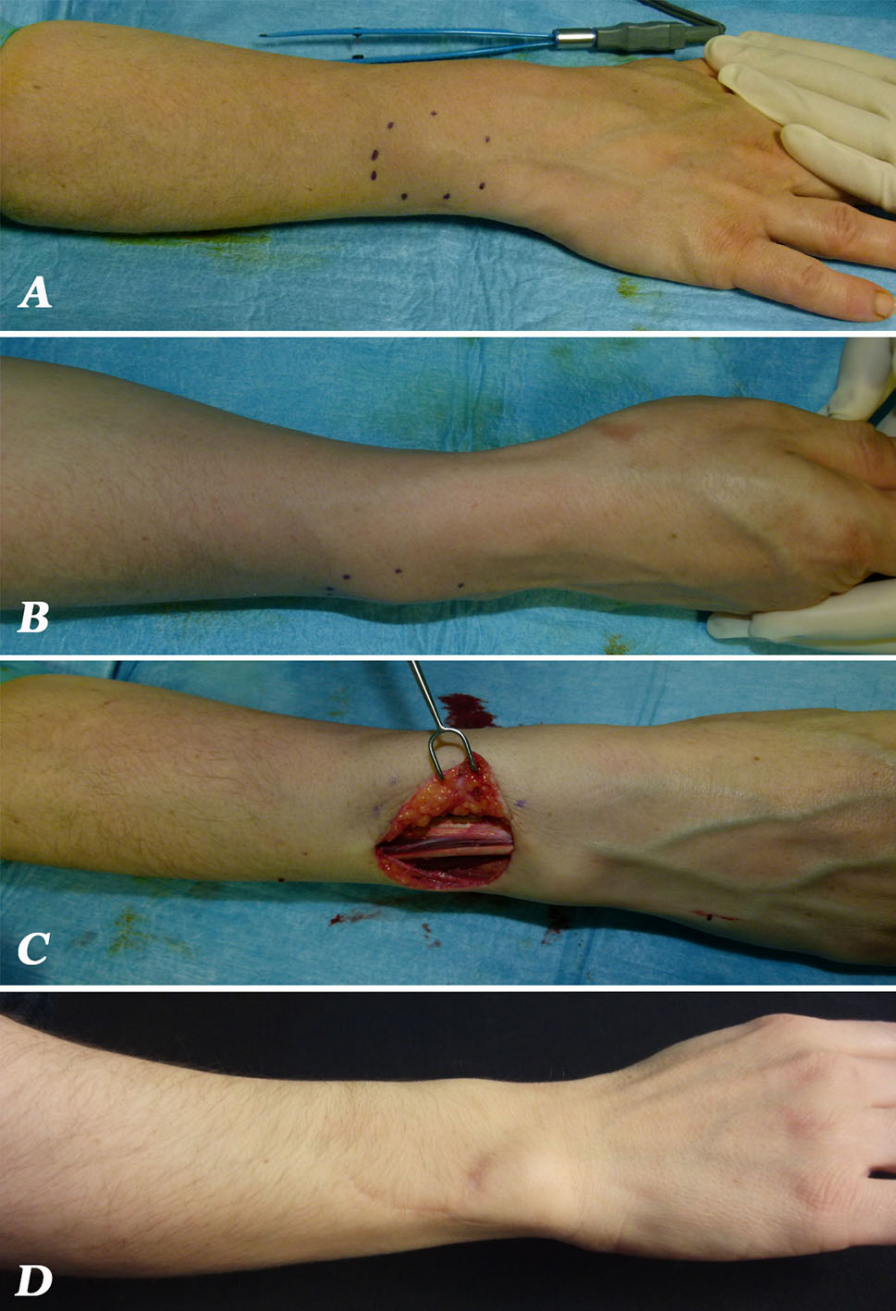
Clinical features of recurrence. **a** Dorsal view with evidence of 4-cm diameter subcutaneous non-solid mass. **b** Radial view with painless swelling proximal to the ulnar styloid. **c** View after complete excision of the mass with evidence of extensor ulnaris carpi and extensors digitorum. **d** Follow-up at 12 months showed no evidence of further recurrence

**Fig. 2:**
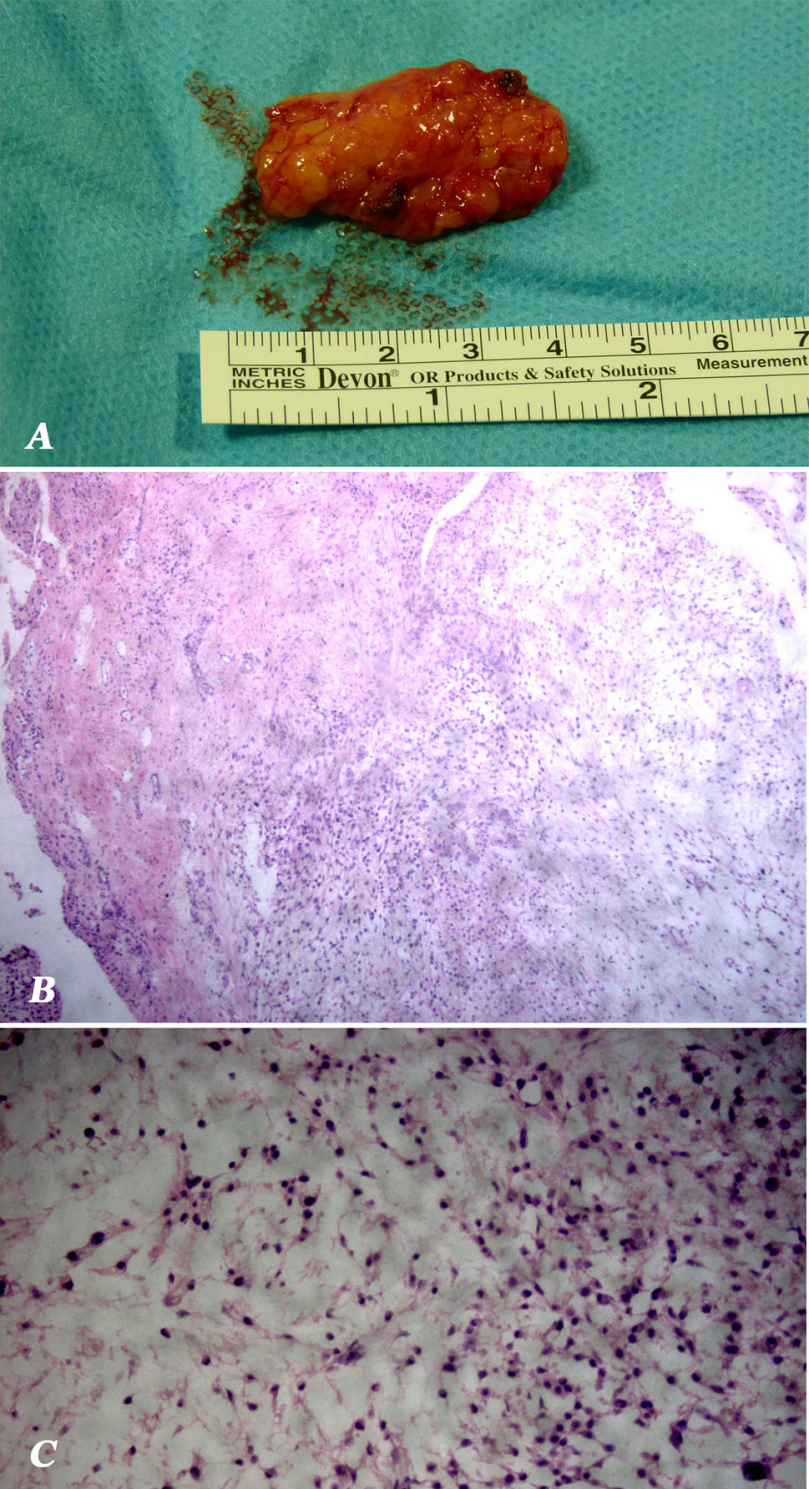
Histology features. **a** En bloc excision of the mass after recurrence. **b** Mixed form of neurothekeoma with lobules composed by cells in part fusal, in part epithelial and in part giant in a background of abundant myxoid stroma (hematoxylin and eosin, magnification ×40). **c** Higher magnification section of mixed neurothekeoma (hematoxylin and eosin, magnification ×200)

A close follow-up was performed; at 3 months a new subcutaneous lesion in the dorsal compartment was detected (Fig. 1a, b) and scanned with ultrasonography. The patient underwent revision surgery with dorsal and proximal extension of the previous scar; the subcutaneous lesion appeared surrounded by scar tissue and adherent to the tendon sheath (Fig. 1c). Histologically, a recurrence of neurothekeoma, with discrete myxoid stroma and cellular elements in part epithelial and in part giant, was found (Fig. 2). With immunohistochemistry there was positivity only to vimentin; S100-protein, EMA and CD10 while cytokeratin AE1-3, CD99, CD34, actina and NSE were all negative. There was a mitotic activity of 4 % (Ki67). All margins were negative.

There was no recurrence by the 12-month follow-up, according to magnetic resonance evaluation (Fig. 1d).

## Discussion

Neurothekeoma is a rare and benign tumor of the nerve sheath first described as being like nerve sheath myxoma by Harkin and Reed in 1969 [6] and subsequently termed neurothekeoma by Gallagher and Helwig in 1980 [7].

It typically presents as a benign, asymptomatic, solitary, slow growing and dome-shaped lesion that only rarely ulcerates the skin. Neurothekeoma preferentially affects the dermis of the cervicofacial areas and shoulders of young women. In our case the lesion was located on the wrist in the subcutaneous tissue near to the sixth extensor compartment. While most have been located in the dermis, the literature describes a few cases where it has arisen in the mouth, the nose, the cranium, the cauda equine, the spinal canal, within the peripheral nerves, the cerebellopontine angle, the mediastinal, the hypopharynx and the external auditory canal [8–14]. To the best of our knowledge, this is the first case report of neurothekeoma of the wrist developing in the synovial tissue in an adult female.

Usually nerve sheath myxoma does not have aggressive local growth, does not have a tendency to metastases, and recurrences are very uncommon [8]. Our group described a recurrence after 3 months probably due to the atypical site of localization or to the histologic and immunohistochemical characteristics.

In fact several studies have classified nerve sheath myxomas into three groups based on cellularity, mucin content and growth pattern: a hypocellular or myxoid type, a hypercellular type and a mixed type [8, 15].

The hypocellular group consists of well-circumscribed multilobulated tumors usually located in the reticular dermis and often extending into the subcutaneous fat with a prominent myxoid stroma and positive to vimentin and S100-protein. According to Laskin et al. [16] the myxoid type of neurothekeoma showed neurosustentacular differentiation (glial cell, Schwann cell and melanocyte) and is the bona fide nerve sheath tumor.

The cellular types are composed of ill-defined nests and fascicles of cells, rather scant mucin and are negative to S100 and positive to vimentin and melanoma specific antigen (NK1/C-3). According to Laskin et al. [16] cellular and mixed type neurothekeoma failed to show convincing evidence of neurosustentacular differentiation and, thus, warranted a separate classification. Rarely the tumor may demonstrate an infiltrative growth pattern, brisk mitotic activity and cytological pleomorphism. However, these atypical features do not influence the prognosis of the tumor [17].

Based on these conisdertaions, mixed type and hypercellular type could show unusual local recurrence because of the presence of cells with mitotic activity; nevertheless a complete surgical excision with the tumor margins free, the clinician has to consider this eventuality. Moreover, Laskin et al. [16] noticed that myxoid/hypocellular neurothekeomas occurred more commonly in male patients (male:female ratio 6:5) in the fourth decade and were found on both the upper and lower limbs. In contrast, the cellular/mixed group affected more females (male:female ratio 4:7) in the second decade with an upper body distribution [16].

Generally, neurothekeomas are difficult to diagnose prior to performing a biopsy, due to the lack of specific clinical manifestations or imaging characteristics. In fact in our case the diagnosis of wrist mass was oriented towards synovial cyst prior to surgical excision. The differential diagnosis of neurothekeoma should include other neural entities, such as schwannoma, true neuroma and myxoid neurofibroma. The effective treatment of choice is complete surgical excision with clear margins. No malignant transformation or metastases have been reported and local recurrence is extremely uncommon, but possible, especially for the cellular/mixed group [16, 17]. Although this is a rare type of tumor, the clinician should consider this entity in differential diagnosis, as it is imperative to distinguish it from malignant lesions, in order to avoid unnecessary aggressive treatment. Faced with a suspected diagnosis is therefore advisable to perform a punch biopsy of the lesion to determine with certainty the correct path of the therapeutic procedure.

Basically, our case report adds an important element in the correct clinical management of neurothekeomas: faced with a histological diagnosis with an unusual localization and mixed or hypercellular type, clinicians must consider the possibility of an early local recurrence, suggesting a close clinical and radiological follow-up.
